# On Well-Being: Current Research Trends And Future Directions

**DOI:** 10.4103/0973-1229.40564

**Published:** 2008

**Authors:** C. Robert Cloninger

**Affiliations:** * *Wallace Renard Professor of Psychiatry, Genetics and Psychology; Director, Sansone Center for Well-Being*

## Introduction

Perspectives that lead to war, greed, and divisive propaganda dominate the world stage at present. Despite this, there is hope for greater well-being if people begin to express what we now know about the sources of peace, love, and happiness. For many years the science of well-being was limited by uncertainty about valid measures and by uncertainty about effective methods of treatment. Now both of these deficiencies are being effectively addressed by a fundamental understanding of the nature of well-being and its development at the levels of individuals, societies, and the world as a whole.

It is now clear that well-being can be measured by four convergent methods: the presence of (1) positive emotions and the absence of negative emotions, as measured by the Positive and Negative Affect Scale (PANAS) (Watson and Clark, 1997); (2) mature character traits, including self-directedness, cooperativeness, and self-transcendence, as measured by the Temperament and Character Inventory (Cloninger, Svrakic and Przybeck, 1993); (3) life satisfaction or quality of life, as measured by the Satisfaction With Life Scale (SWLS) (Pavot and Diener, 1993), and (4) character strengths and virtues, such as hope, compassion, and courage (Peterson and Seligman, 2004). Research has shown that these measures converge to identify the same individuals, which means that authentic well-being involves all of these features. In other words, a person cannot feel good (as measured by positive emotions and life satisfaction) without doing good (as measured by maturity of character and virtuous conduct) (Cloninger, 2004).

## Features that Characterize Well-Being Only Develop as a Result of Growth in Self-Awareness

However, to cultivate well-being we must understand its actual source, rather than try and imitate it superficially. Skills training or practicing particular forms of socially respectable coping behaviours might produce that which could be called virtue by behaviorally oriented therapists. However, such behavioural skills provide little or no benefit in promoting hope or positive emotions and are quickly extinguished when the reinforcement is no longer maintained consistently (Linehan, Heard and Armstrong, 1993). Likewise, practicing laughter and good cheer has little or no benefit in promoting character strengths or virtues until a person becomes aware for himself or herself of what he or she has to be grateful and cheerful about spontaneously. In other words, individual features of well-being can be imitated superficially, but the convergent set of features that characterize well-being (positive emotions, character strengths, virtues and life satisfaction) only develops as a result of growth in self-awareness (Cloninger, 2004). The deliberate struggle in cognitive behavioural therapy to be rational and to suppress desires is hard work and leads to frequent drop outs, relapses, and recurrence of illness, because the underlying perspectives on life that make a person vulnerable to ill-health have not been transformed. Some forms of experiences with behaviour and/or thought may help a person to be aware, but cognitive behavioural interventions alone have little or no benefit without growth in self-awareness. Learning to face and accept the reality of the present moment in self-awareness is the key to authentic well-being.

Recent research has shown that augmentation of cognitive behavioural therapy with increased awareness of one's body, mind, or spirit reduces relapse and recurrence rates in therapy for a wide range of disorders, as compared to cognitive behavioural therapy alone (Cloninger, 2006b; D'Souza and Rodrigo, 2004; Fava *et al.*, 2005; Teasdale *et al.*, 2002). Well-being depends on the integration of all three aspects of human beings—their body, thoughts, and psyche — in self-awareness. Unfortunately, most therapies are highly focused on behaviour or thought and seldom address spiritual issues or the integration of all three aspects of being. Future work on the integration of all these is strongly needed.

For example, positive psychology has been preoccupied with the cognitive and social aspects of well-being, neglecting both its neurobiological and its spiritual roots (Cloninger, 2005). However, research now shows that self-transcendence has a strong neurobiological basis in human evolution, with unique genetic determinants (Gillespie *et al.*, 2003) and serotonergic neurotransmission (Borg *et al.*, 2003). Self-transcendence is also important for the preservation of grey matter for meta-cognition as people age (Kaasinen, 2005). Thus, it is clear that well-being depends on the integration of all the three aspects of human beings (body, thoughts, and psyche).

## Personality Disorders and Well-Being

Current trends in research and treatment of personality disorders are in great need of a fundamental change in direction as a result of what has been learned about well-being. Earlier Western concepts of personality disorder, as now reified in the American Psychiatric Association's Diagnostic and Statistical Manual, are defined primarily by poor development of self-directedness. A mature person is supposed to be simply someone who is self-directed – that is, responsible, purposeful, and resourceful. To a lesser extent, society also expects people to be cooperative, that is, to get along with one another by being tolerant, forgiving, helpful, and honest. However, self-transcendence – man's search for what is beyond individual human existence – is also crucial for the development of resilience and maintenance of well-being (Cloninger, 2004). Future work on personality needs to be based on a fuller understanding of the interplay between all three aspects of human character for the development of well-being.

The same dimensions of personality that predict well-being in the general population are also predictive of recovery from major mental disorders. Self-directedness is the major predictor of recovery from schizophrenia, mood disorders and eating disorders (Anderson *et al.*, 2002; Eklund *et al.*, 2004), and treatments targeting cooperativeness and self-transcendence also make important contributions to clinical outcome in randomized controlled trials in a wide range of mental disorders (D'Souza and Rodrigo, 2004).

These results show that the treatment of the positive aspects of mental and physical health is crucial for good clinical outcomes. Psychiatrists have traditionally focused on remission of negative symptoms of disease and reduction of harm, whereas the Recovery Movement has questioned the validity of disease labels and demanded focus on empowerment of patients. Self-directedness is the major indicator of a patient's sense of coherence, self-esteem and personal mastery (Eklund *et al.*, 2004). In my opinion, the perspective of both traditional psychiatry and the Recovery Movement has been too adversarial and narrow: mental disorders are real debilitating syndromes, but psychiatrists and patients need to work together in a mutually respectful manner to facilitate both the development of positive mental health and the reduction of the symptoms of ill-health. What I have found recently is that the predictors of resilience and recovery are personality traits that are partly heritable and partly determined by factors unique to each individual. As a result, my current work is focused on deconstructing traditional clinical syndromes like schizophrenia to understand the epigenetic puzzle of how risk and recovery factors interact in complex ways so that 40% of the monozygotic co-twins of a schizophrenic can be mentally normal (Gottesman and Shields, 1982).

## Reduced Well-Being and Post-Traumatic Stress Disorders

Much has been learned about the precipitants of post-traumatic stress disorders (PTSD). Lack of resilience when challenged by stress is predicted by poor character development, particularly in response to major stresses like those occurring in war or natural disasters (Cloninger, 2004; North *et al.*, 1999). The incidence of PTSD among soldiers in Iraq and Afghanistan is high (at least 15%) and efforts to reduce this by cognitive behavioural therapy have had only weak effects when people are enmeshed for long periods in conditions that are violent and dehumanizing.

The problem of reduced well-being and PTSD associated with aggressive militarism has raised consideration of the societal requirements for well-being. What must society provide to facilitate the well-being of the people who comprise the population? It is already well-established that Western consumerism is not conducive to well-being: people do not grow in life satisfaction with increases in income above what is required for their basic requirements for food, housing, and health care. For example, life satisfaction in the USA has not improved from 1930 to the present, despite the increase in inflation-adjusted income (Myers and Diener, 1996). Furthermore, indicators of medical morbidity and mortality show that countries like the USA and England, whose policies are based on competition, militarism and consumerism, are less healthy than those of countries like Sweden and France that are more focused on public well-being. Countries like India, which are rapidly adopting the Anglo-American focus on competition, militarism and consumerism, would do well to recognize the fundamental need to attend to the well-being of its people in order to sustain a healthy and happy society.

## Basic Conditions for Well-Being

The basic conditions needed for well-being are observable at both the individual and the societal level. For well-being we need to let go of struggles, work in the service of others, and grow in awareness of our inseparable connection to one another and the world of which we are integral parts. Wherever there is a sense of separateness and division, there is fear, which leads to despair, conflict and ill-health. Wherever there is a sense of connectedness and unity, there is hope, love, and freedom to grow in awareness and well-being. The beauty of these contrasts between the conditions of ill-health and well-being is that human beings can and must use their intelligences to learn to face their fears and let go of their conflicts if they wish to grow in self-awareness and well-being. Exploitation, domination and violence can never produce well-being or life satisfaction. As Gandhi said, “When I despair, I remember that all through history the way of Truth and Love has always won. There may be tyrants and murderers and for a time they may seem invincible, but in the end, they always fail. Think it, always.” (Gandhi, 1997).

The requirements for well-being can be fully understood by going beyond both the individual and the societal levels of observation. Recent work emphasizes the sense that human well-being requires a coherent spiritual perspective. Specifically, the foundation for personal well-being is the self-awareness that each being is an inseparable part of a universal unity of being. Fortunately, there is emerging evidence for increased transpersonal awareness, as shown by increasing prevalence of self-transcendent experiences in surveys of English people between 1987 and 2000 (Hay, 2007). David Hay found that 48% of British people reported self-transcendent experiences in 1987, whereas by 2000 the percentage had increased to 76% in population-based surveys. Greater awareness of our transpersonal connections with what is beyond individual human existence is an essential ingredient of well-being. Even agnostics and atheists have a spiritual perspective that helps them to understand the purpose and meaning of their lives (Hay, 2007). Well-being depends on the integration of complex adaptive systems at every level of interaction—within individuals, among individuals, within communities, among communities, within nations, and among nations globally (Cloninger, 2004). To be free and happy, human beings need to realize for themselves that, as Jiddu Krishnamurti often said, “You are the world”: “In oneself lies the whole world and if you know how to look and learn, then the door is there and the key is in your hand. Nobody on earth can give you either that key or the door to open, except yourself.” (Krishnamurti, 1972).

## Therapeutic Intervention and Practical Exercises for Growth in Self-Awareness

Unfortunately, mystics like Krishnamurti have not provided psychotherapists with practical ways to help others develop well-being. He pointed out the goal but did not provide the means to the goal (Shainberg, 1995). By his own admission, few people, if any, were able to follow his advice or example. At the present time, I am developing a therapeutic intervention that provides practical exercises for the body, mind and psyche to facilitate growth in self-awareness (Cloninger, 2006b). The exercises are taught in a treatment programme called *The Happy Life: Voyages to Well-Being*, which involve a series of therapeutic sessions in which I am meeting with a young woman who wants to be happier and more aware. No one can climb a mountain for another, but it is helpful to have a guide point out a path for ascent, with practical means for overcoming obstacles that may be encountered along the way. The materials are being distributed as a set of DVDs with workbooks and study guides, prepared by a non-profit foundation called the Anthropaideia Foundation, to facilitate their use for training mental health professionals, in self-help, and as adjuncts to therapy (Cloninger, 2006a). These materials are available only in the English language at present. They can be used in treatment and for conducting randomized controlled trials for those who are interested. Additional information is available on my website (https://psychobiology.wustl.edu) and the Anthropaideia website (http://aidwellbeing.org). There is a great need for future research to evaluate and adapt such materials for the promotion of well-being around the world.

## Concluding Remarks

Much progress has been made in the science of well-being during the past decade. There is now consensus on valid measures of well-being. Treatment methods have been developed and supported by the findings of randomized controlled trials, drawing on work from several research areas, including psychodynamic psychiatry, positive psychology, and research on the development of personality and emotionality. The basic conditions for well-being have been described in ways that are concordant at several levels of observation – personal, societal, and spiritual. This work has been found to be important for people with any form of psychopathology and also for the flowering of positive health in the general population. However, much more work is needed to be able to improve the health of people with serious mental disorders consistently and to help the majority of people to learn how to live fulfilling lives. Many influences within the mental health profession and in society at large are resistant to incorporating what has been learned, largely due to lack of awareness. Growth in self-awareness will hopefully allow the principles of well-being to be more accessible to everyone in society, and this is especially necessary today because of the challenges posed by globalization.

### Take Home Message

The practices that lead to well-being are well established – letting go of all struggles, working in the service of others, and growing in awareness. The same principles operate at the level of individuals and in society as a whole. There is great hope for improved levels of life satisfaction, peace and happiness in the world as people grow in awareness of the effective means to well-being.

## About the Author

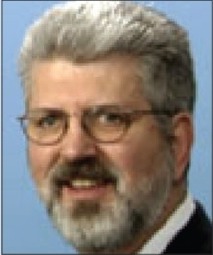



C. Robert Cloninger is Wallace Renard Professor of Psychiatry; Professor of Psychiatry, Genetics, and Psychology; and Director, Center for Psychobiology of Personality, Washington University. He is a world leader in studies of the relation of genes to mental illness. He has developed a compelling theory of psychobiology. Current work involves studies of normal and abnormal personality, alcoholism, schizophrenia and related traits. He is also on the Honorary International Editorial Board of MSM.
